# Highlight report: Cell type selection for toxicity testing

**DOI:** 10.17179/excli2018-2017

**Published:** 2018-12-18

**Authors:** H. M. Bolt

**Affiliations:** 1 IfADo - Leibniz Research Centre for Working Environment and Human Factors, Dortmund, GERMANY

## ⁯⁯⁯⁯

An important step in *in vitro* test system development is the choice of an adequate cell line which depends on the intended application of the assay. In a recent study, Tuuli Karhu and colleagues from Helsinki University compared a set of cell lines for their susceptibility towards eight GATA4 targeting compounds (Karhu et al., 2018[7]). GATA4 is a transcription factor involved in cardiac development (Gupta et al., 2013[6]; Kikuchi et al., 2010[8]; Rysä et al., 2010[15]; Pikkarainen et al., 2004[13]). The goal of the study was to identify which cell line allows the most sensitive cytotoxicity screening of these compounds. The tested cell lines included the myoblast cell line H9c2 established from rat myocardium; primary neonatal rat cardiac fibroblasts; mouse embryonic fibroblasts; mouse embryonic stem cells (mECSs), mouse embryonic stem cell derivatives from day 5 embryoid bodies; induced pluripotent human stem cells (hiPSC); and hiPSC-derived cardiomyocytes. The most susceptible cell lines towards the set of test compounds were hiPSC and mESC, while cardiomyocytes, fibroblasts and H9c2 cells were most resistant (Karhu et al., 2018[7]). Of course screening for the most sensitive cell line does not guarantee that the test cells will be most relevant for the human *in vivo* situation. However, if one is interested in a cytotoxicity screening system with the highest sensitivity, the recommendation of the authors to further use hiPSC seems reasonable.

In recent years, the development of stem cell based test systems has been a major focus of research (Leist et al., 2017[10]; Godoy et al., 2013[3]; Krug et al., 2013[9]). The most frequently applied strategy is to expose stem cells to test compounds, when they differentiate to more mature cell types (Shinde et al., 2017[17]; Pallocca et al., 2016[12]).

This approach has been used for developmental neurotoxicity (Waldmann et al., 2014[18]; Meganathan et al., 2015[11]; Weng et al., 2014[19]; Rempel et al., 2015[14]) and for cardiotoxicity (Chaudhari et al., 2016[1][2]; Sampaio et al., 2016[16]) testing. While tests that analyze the influence of compounds on the differentiation process are already successfully applied, it still remains a challenge to generate mature cell types, e.g. hepatocytes that closely resemble the primary cells in an adult organ (Godoy et al., 2016[5], 2018[4]). Although much progress has been achieved in stem cell based test system development, systematic analysis of human *in vivo* relevance still remains a major challenge.
